# Resistance to systemic immune checkpoint inhibition in the peritoneal niche

**DOI:** 10.1136/jitc-2022-004749

**Published:** 2022-06-21

**Authors:** Daryl Kai Ann Chia, Yong Xiang Gwee, Raghav Sundar

**Affiliations:** 1 Department of Surgery, University Surgical Cluster, National University Hospital, Singapore; 2 Department of Haematology-Oncology, National University Cancer Institute, Singapore; 3 Yong Loo Lin School of Medicine, National University of Singapore, Singapore; 4 Cancer and Stem Cell Biology Program, Duke-NUS Medical School, Singapore; 5 The N.1 Institute for Health, National University of Singapore, Singapore; 6 Singapore Gastric Cancer Consortium, Singapore

**Keywords:** Immunotherapy, Tumor Microenvironment, Gastrointestinal Neoplasms

## Abstract

Immune checkpoint inhibition (ICI) is an established therapeutic option for patients with deficient mismatch repair or high levels of microsatellite instability tumors. Yet, response to ICI among this group is varied, with nearly one-third of patients exhibiting primary resistance. Initial efforts in studying mechanisms of resistance to ICI have focused on intrinsic tumor factors. Host factors such as metastatic niches have unique biological properties that may mediate resistance to ICI but have been less studied date. Patients with metastatic d-MMR/MSI-H gastrointestinal cancers and peritoneal metastases (PM) who had concurrent ascites have been recently shown to have worse outcomes with ICI therapy compared with patients with PM without ascites and patients with non-PM metastases. The juxtaposition of tumors with an intrinsic sensitivity to ICI failing to respond by virtue of the presence of ascites within the peritoneum, brings to the forefront the critical role of the metastatic niche. In this commentary, we discuss mechanisms for ICI resistance that may arise from the immunoprivileged state of the peritoneal cavity, paracrine factors within malignant ascites or tumor-peritoneum interactions. An improved understanding of the peritoneal microenvironment and the use of peritoneal-directed therapies may ameliorate the modest benefit of ICIs in this unique clinical entity.

The dramatic and sustained responses of tumors to immune checkpoint inhibition (ICI) have led to a paradigm shift in the management and treatment of cancer. Patients with advanced cancers who were previously considered incurable have reported dramatic, and more importantly, sustained responses to ICI therapy, a phenomenon rarely seen in those treated in with chemotherapy and targeted therapy. However, as these deep and durable responses are not universal, predictive biomarkers of sensitivity to ICI play an important role in patient selection and therapy choice. High levels of microsatellite instability (MSI-H) through deficient mismatch repair (d-MMR) leads to frameshift mutations and generation of multiple foreign amino acid sequences that are novel antigens to the host immune system. A high tumor mutation load results in increased immunogenicity and exquisite sensitivity to ICI. MSI-H tumors arise due to germline mutations in one of the DNA mismatch repair genes or through somatic promoter hypermethylation of *MLH1* and are detected across multiple tumor types including gastrointestinal (colorectal, gastric, hepatobiliary) and endometrial cancer.^1^ The identification of a predictive biomarker to systemic therapy (pembrolizumab, anti-PD-1 checkpoint inhibitor), across multiple tumor types based on a genomic signature led to the Food and Drug Administration (FDA, United States of America) granting its first ever tissue-agnostic approval for the treatment of deficient mismatch repair instability (dMMR)/MSI-H tumors.^2^ However, even among this group of ICI-sensitive tumors, there exists a spectrum in the degree of responsiveness, with almost a third of MSI-H tumors potentially demonstrating up-front primary resistance to single-agent anti-PD-1 inhibition.^3^

A number of mechanisms have been implicated in the resistance of MSI-H tumors to ICIs. These include tumor intrinsic mechanisms such as antigen presenting defects through loss of β2 microglobulin and aberrations in immune-response-related genes. Factors within the surrounding tumor microenvironment include the diminished repertoire of cytotoxic T-cells, metabolites such as lactate resulting in abrogation of T-cell activity, upregulation of FOXP3+ regulatory T-cells mediated through TGF-β pathways, and recruitment of myeloid-derived suppressor cells.^4^ This framework was further validated in preclinical mouse models of ovarian cancer with peritoneal metastases (PM) demonstrating reversal of epithelial-mesenchymal-transition (EMT), decreased immune exclusion and better response when chemotherapy was given in conjunction with a TGF-β blockade.^5^ Similarly, peritoneal administration of Toll-like receptors 7/8 agonists exhibited potent antitumor activity against PM mediated by a decrease in Foxp3+ T-regulatory cells in mouse model of CRC.^6^ While MSI-H tumors generate a high mutational burden, the quality of mutations generated also plays an important role; a larger proportion of insertion-deletion (indel) mutations compared with missense mutations has been shown to have increased levels of T-lymphocyte infiltration and better response to anti-PD-1 therapy.^7^ While most studies have focused on intrinsic tumor factors, few have looked at host factors, in particular, metastatic niches with unique biological properties that mediate resistance to ICI.

The peritoneum, the largest of three serous cavities in the human body (along with the pleura and pericardium) is an immunological niche regulated by several interconnected pathways and networks and is thought to be sequestered from systemic circulation by the peritoneal–plasma barrier, limiting access to chemotherapy and the immune system.^8^ Fastric and colorectal primary tumors have a propensity to metastasize to the peritoneum, arising from a cascade of events starting with shedding of tumor cells, trans-coelomic circulation, mesothelial adhesion, submesothelial invasion, facilitated by EMT and systemic metastases via transperitoneal lymphatics.^9^ This is aided by a favorable microenvironment, or premetastatic niche, within the peritoneal cavity through tumor-derived secreted factors, cytokines and exosomes, recruitment of bone-marrow derived cells and tumor-induced host stromal changes including fibroblast alterations and changes in extracellular matrix.^10^ Factors that favor development of premetastatic niches include immunosuppression, inflammation, angiogenesis and vascular permeability, lymphangiogenesis, preponderance for metastases to specific organs also known as organotropism and reprogramming at the metabolic, stromal and molecular level.^11^ Stromal cells within the peritoneum may also facilitate PM by inducing an immunosuppressive microenvironment, for example, through Wt1+ mediated exclusion of peritoneal macrophages and Tim-4+ macrophages impairing antitumor cytotoxic T-cells.^12 13^ In addition, paracrine factors including cytokines, chemokines and growth factors present within malignant ascites contribute to a tumorigenic environment for PM to occur. For example, IL-6 has been detected in malignant ascites in patients with metastatic gastric cancer (mGC) and ovarian cancer, which can induce cell growth and chemoresistance. These mechanisms create an immune-privileged niche for PM and may lead to resistance to ICIs.

To study the clinical implications of metastatic tumors to the peritoneal niche and their sensitivity to ICI, Fucà *et al* examine a multicenter cohort of d-MMR/MSI-H that included 502 metastatic colorectal cancer (mCRC) and 59 mGC patients and report significantly poorer survival in patients with PM and ascites compared with patients with PM without ascites and patients with other non-PM sites of metastases.^14^ In addition, mCRC patients receiving dual ICI (with anti-CTLA-4 and PD-1) therapy trended to better OS irrespective of site/type of metastases. In contrast, mCRC patients receiving mono ICI therapy (anti-PD-1) alone had much poorer survival outcomes. While limited by its retrospective design and relatively small number of patients for subgroup analysis, the study implies that the benefits of ICIs as monotherapy are limited in patients with PM and concurrent ascites despite being d-MMR/MSI-H tumors. The juxtaposition of tumors with an intrinsic sensitivity to ICI (dMMR/MSI-H) failing to respond by virtue of the presence of ascites within the peritoneum, brings to the forefront the critical role of the metastatic niche.

Although MSI-H tumors are sensitive to ICI, they comprise only a small fraction (<5%) of patients with metastatic colorectal and gastric cancer. To date, there is no approval or indication for the use of ICI in MSS mCRC. In comparison, ICIs have modest benefit in MSS mGC when used as a single-agent the third line therapy, but perhaps performs better in combination with chemotherapy as part of first-line therapy. However, even in MSS tumors, patients with PM did not appear to derive benefit from ICI.^15 16^ These data provide orthogonal support for the hypothesis of an immunoresistant PM niche. Additionally, discordance has been described between MSI-H primary tumors and MSS PM.^17^ While the resistance of mGC PM to immunotherapy is more apparent, the benefit of other systemic therapies such as chemotherapy and targeted therapy in this subgroup of patients is controversial. Randomized controlled trials evaluating various systemic therapies including cisplatin plus S-1, ramucirumab (alone or in combination with paclitaxel), TAS-102 (Trifluridine/Tipiracil) and nivolumab monotherapy demonstrated that while patients with mGC PM benefit from such agents, similar to patients with non-PM metastatic GC, the extent of survival benefit was lower in patients with mGC PM, thereby highlighting the significance of PM as a negative prognostic marker. In this regard, there is great potential in integrating locoregional therapy with ICIs, given that the direct cytotoxic effects of intraperitoneal chemotherapy may be potentiated by immunogenic cell death which renders both MSI-H and MSS PM to become susceptible to the host immune system. For instance, the ongoing PIANO study combines Pressurized Intraperitoneal Aerosol Chemotherapy oxaliplatin with systemic nivolumab in patients with GCPM (NCT03172416).

In summary, while MSI-H tumors generally demonstrate good response to ICIs, PMs may demonstrate resistance that arise either from factors pertaining to tumor characteristics, the immunosuppressive state of the peritoneal cavity, paracrine factors within malignant ascites or tumor-peritoneum interactions. A deeper understanding of molecular subtypes of PM, the peritoneal microenvironment and the use of peritoneal-directed therapies will ameliorate the modest benefit of ICIs in this unique clinical entity.
